# Regulation and prognostic relevance of serum ghrelin concentrations in critical illness and sepsis

**DOI:** 10.1186/cc9029

**Published:** 2010-05-25

**Authors:** Alexander Koch, Edouard Sanson, Anita Helm, Sebastian Voigt, Christian Trautwein, Frank Tacke

**Affiliations:** 1 Department of Medicine III, RWTH-University Hospital Aachen, Pauwelsstrasse 30, 52074 Aachen, Germany

## Abstract

**Introduction:**

Ghrelin has been recently identified as a mediator of various beneficial effects in animal models of sepsis. At present, no data are available concerning specific properties of ghrelin in critically ill patients from large cohorts. In order to identify possible pathogenic functions of ghrelin in critically ill patients and human sepsis from a clinical point of view, we aimed at analyzing ghrelin serum concentrations in a large cohort of well characterized patients with critical illness.

**Methods:**

A total of 170 critically ill patients (122 with sepsis, 48 without sepsis) were studied prospectively on admission to the Medical intensive care unit (ICU) and compared to 60 healthy controls. Careful assessment of clinical data, various laboratory parameters, metabolic and endocrine functions as well as investigational inflammatory cytokine profiles have been performed, and patients were followed for approximately three years.

**Results:**

Ghrelin serum concentrations are elevated in critically ill patients as compared to healthy controls, but do not differ between sepsis and non-sepsis patients. The underlying etiologies of critical diseases are not associated with ghrelin serum levels. Neither pre-existing diabetes mellitus nor body mass index is correlated to serum ghrelin concentrations. Ghrelin is not correlated to markers of inflammation or hepatic function in critically ill patients. In the subgroup of non-sepsis patients, ghrelin correlates inversely with renal function and markers of carbohydrate metabolism. High ghrelin levels are an indicator for a favourable prognosis concerning mortality at the ICU in sepsis patients. Furthermore, ghrelin is significantly associated with the necessity of ventilation in critically ill patients.

**Conclusions:**

Ghrelin serum concentrations are elevated in all circumstances of critical disease, including sepsis and non-sepsis patients. High ghrelin levels are a positive predictor of ICU-survival in sepsis patients, matching previous results from animal models. Future experimental and clinical studies are needed to evaluate ghrelin as a novel prognostic tool in ICU patients and its potential therapeutic use in sepsis.

## Introduction

Human ghrelin, a 28-amino acid peptide, is predominantly synthesized by the stomach and is the only identified endogenous ligand for the growth hormone secretagogue receptor 1a (GHS-R1a) [1]. GHS-R1a is expressed in various tissues in different concentrations and has been found in pituitary, hypothalamus, heart, blood vessels, lung, pancreas, intestine, kidney, adipose tissue, B- and T-cells and neutrophils [2-4]. This wide distribution of GHS-R1a suggests multiple roles of ghrelin with regard to cerebral, renal and pulmonary function, hemodynamics, gut barrier and immune system. Nevertheless, about two third of the circulating ghrelin is derived from the stomach and nearly all of the remaining one third from the small intestine [5,6]. Rat ghrelin is very similar to human ghrelin and differs only by two amino acids [7]. Therefore animal models have been widely used to investigate potential functions of ghrelin. Ghrelin stimulates growth hormone secretion in rats and humans, regulates food intake and energy homeostasis and has vasodilatatory effects as a physiological antagonist of endothelin-1 [8-10]. Moreover, protective effects of ghrelin in animal sepsis models have been reported. Specifically, ghrelin was found to mediate improvement of tissue perfusion in severe sepsis [11], down-regulation of proinflammatory cytokines in sepsis through activation of the vagus nerve [12], stabilization of gut barrier function in sepsis [13], attenuation of sepsis-induced acute lung injury [14] and protection against endotoxemia-induced acute kidney failure [15]. On the basis of these findings GHS-R1a has been regarded as a possible drug target in critical care medicine with ghrelin or a ghrelin mimetic as a new therapeutic option [16].

Nevertheless, the findings on the responses of ghrelin to endotoxin in animal models and in healthy humans are partially contradictory and inconsistent. Beyond that, there are no data concerning the mechanisms of regulation in critically ill patients from large cohorts. Before testing the possible therapeutic effects of ghrelin in humans, clinical studies on profiles of endogenous ghrelin regulation in the critically ill have been demanded [17]. Up to now, there has been only a small pilot study with 16 ICU patients, reporting low initial, but, during ICU treatment, increasing ghrelin serum concentrations [18]. The present study was conducted with a large cohort of well characterized critically ill patients to provide information on ghrelin serum concentrations in different circumstances of critical disease, to identify possible pathogenic functions of ghrelin by correlations with a wide number of markers of inflammation, organ dysfunction and metabolism and to examine potential protective effects of ghrelin in critically ill patients and human sepsis from a clinical point of view.

## Materials and methods

### Study design and patient characteristics

The present study was approved by the local ethics committee. Before inclusion, written informed consent was obtained from the patient, his or her spouse or the appointed legal guardian. We studied 170 patients (111 male, 59 female with a median age of 62 years; range 18 to 86 years) (Table 1) [19]. Patients were included consecutively upon admission to the Medical ICU of the RWTH University Hospital Aachen due to critical illness. Patients were excluded from this study, if they were expected to have a short-term (<72 h) intensive care treatment, for example, due to post-interventional observation or acute intoxication. All patient data, clinical information and blood samples were collected prospectively.

**Table 1:** Baseline patient characteristics and ghrelin serum concentrations

Parameter	All patients	Sepsis	non-Sepsis
Number	170	122	48
Sex (male/female) n	111/59	81/41	30/18
Age median (range) (years)	63 (18 to 86)	64 (20 to 86)	60 (18 to 79)
APACHE-II score median (range)	14 (0 to 31)	14 (0 to 31)	15 (0 to 31)
SAPS2 score median (range)	44 (0 to 80)	45 (0 to 79)	41 (13 to 80)
ICU days median (range)	8.5 (1 to 137)	10 (1 to 137)	6 (1 to 45)
Hospital days median (range)	27 (2 to 151)	30 (2 to 151)	14 (2 to 85)
Death during ICU n(%)	54 (32%)	42 (34%)	12 (25%)
Death during follow-up n(%)	88 (52%)	64 (53%)	24 (50%)
30-day mortality [%]	32%	32%	31%
60-day mortality [%]	39%	40%	35%
90-day mortality [%]	42%	44%	35%
180-day mortality [%]	45%	48%	38%
1-year mortality [%]	49%	51%	44%
Mechanical ventilation n(%)	113 (67%)	82 (67%)	31 (65%)
Ventilation time median (range) [h]	66 (1 to 2,966)	127.5 (1 to 2,966)	31 (1 to 755)
pre-existing diabetes n(%)	56 (33%)	39 (32%)	17 (35%)
BMI median (range) (m²/kg)	25.8 (14.0 to 59.5)	26.0 (14.0 to 59.5)	25.1 (17.5 to 53.3)
Serum IGF-1 median (range) (μg/L)	54 (25 to 295)	54 (25 to 295)	49 (25 to 165)
Serum growth hormone median (range) (μg/L)	1.5 (0.1 to 128.0)	1.3 (0.1 to 128.0)	2.0 (0.1 to 22.3)
Serum ghrelin median (range) (pmol/L)	18.4 (5.0 to 129.5)	18.4 (5 to 113.8)	18.4 (5 to 129.5)

APACHE: acute physiology and chronic health evaluation; BMI: body mass index; IGF-1, insulin-like growth factor-1; SAPS: simplified acute physiology score

Blood samples of 60 healthy non-diabetic blood donors (33 male, 27 female, with a median age of 46 years; range 31 to 58 years) with normal values for blood counts, C-reactive protein and liver enzymes have been examined as a control group.

### Characteristics of sepsis and non-sepsis patients

A total of 122 of the 170 critically ill patients (72%) enrolled in this study, fulfilled the criteria of bacterial sepsis, according to the American College of Chest Physicians and the Society of Critical Care Medicine Consensus Conference Committee for severe sepsis and septic shock [20]. In the majority of sepsis patients the identified origin of infection was pneumonia (Table 2). Non-sepsis patients were admitted to the ICU due to cardiopulmonary disorders (myocardial infarction, pulmonary embolism, and cardiac pulmonary edema), decompensated liver cirrhosis or other critical conditions and did not differ in age or sex from sepsis patients. As expected, significantly higher levels of laboratory indicators of inflammation (that is, C-reactive protein, procalcitonin, white blood cell count) were found in sepsis patients than in non-sepsis patients (Table 1, and data not shown). Both groups did not differ in acute physiology and chronic health evaluation (APACHE II) score, vasopressor demand, or laboratory parameters indicating liver or renal dysfunction (data not shown). ICU-mortality of all critical care patients was 32%, and 52% of the total initial cohort died during the overall follow-up of 900 days (Table 1).

**Table 2:** Disease etiology of the study population

	sepsis	non-sepsis
	n = 122	n = 48
**Etiology of sepsis critical illness**		
Site of infection n (%)		
Pulmonary	72 (59%)	
Abdominal	22 (18%)	
Other	28 (23%)	
**Etiology of non-sepsis critical illness**		
n (%)		
Decompensated liver cirrhosis		17 (35%)
Cardiopulmonary diseases		18 (38%)
Other		13 (27%)

### Comparative variables

The patients in the sepsis and non-sepsis groups were compared by age, sex, body mass index (BMI), pre-existing diabetes mellitus and severity of disease using the APACHE II score upon admittance to the ICU. Careful recording of intensive care treatment, such as volume therapy, vasopressor infusions, demand of ventilation and ventilation hours, antibiotic and antimycotic therapy, renal replacement therapy and nutrition, has been performed. Additionally, a large number of laboratory parameters that were routinely assessed during intensive care treatment have been analyzed.

### Quantification of ghrelin, IGF-1 and growth hormone serum concentrations

Peripheral venous blood samples were obtained at admission before therapeutic intervention, immediately placed on ice, centrifuged and stored at 80°C. All patients had been fasting for at least three hours before admission to the ICU. All measurements were performed in a blinded fashion. Ghrelin serum concentrations were measured using an enzyme-linked immunosorbent assay (ELISA) according to manufacturer's instructions (Millipore, Schwalbach, Germany). Furthermore, growth hormone (Immulite 2000 hGH, Siemens, Erlangen, Germany) and IGF-1 (Immulite 2500 IGF-1, Siemens, Erlangen, Germany) were measured by chemiluminescent immunometric assay in the routine clinical laboratory.

### Statistical analysis

Due to the skewed distribution of most of the parameters in critically ill patients, data are given as median and range. Differences between two groups were assessed by Mann-Whitney-*U*-test and multiple comparisons between more than two groups have been conducted by Kruskal-Wallis-ANOVA and Mann-Whitney-*U*-test for post hoc analysis. Box plot graphics were employed to illustrate comparisons between subgroups. They display a statistical summary of the median, quartiles, range and extreme values. The whiskers extend from the minimum to the maximum value excluding outside and far out values which are displayed as separate points. An outside value (indicated by an open circle) is defined as a value that is smaller than the lower quartile minus 1.5-times interquartile range, or larger than the upper quartile plus 1.5-times the interquartile range. A far out value is defined as a value that is smaller than the lower quartile minus three times the interquartile range, or larger than the upper quartile plus three times the interquartile range. All values, including *outliers*, have been included for statistical analyses [19]. Correlations between variables have been analysed using the Spearman correlation tests, where values of *P *<0.05 were considered statistically significant. The prognostic value of the variables was tested by univariate and multivariate analysis in the Cox regression model. Kaplan Meier curves were plotted to display the impact on survival. All statistical analyses were performed with SPSS version 12.0 (SPSS, Chicago, IL, USA).

## Results

### Ghrelin serum concentrations are elevated in critically ill patients as compared to healthy controls and are not different between sepsis or non-sepsis patients

**Figure 1:**
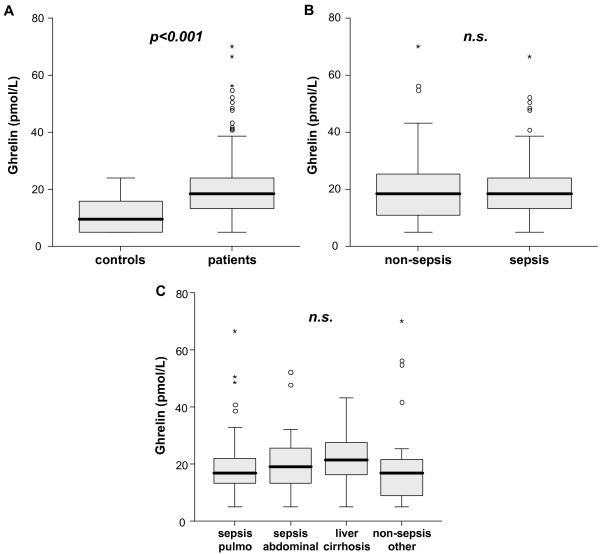
**Serum ghrelin concentrations in critically ill patients**. **(A) **Serum ghrelin levels are significantly (*P *<0.001, U-test) elevated in critically ill patients (n = 170) as compared to healthy controls (n = 60). **(B) **No significant differences are detected between ICU patients with sepsis and non-septic etiology of critical illness. **(C) **Ghrelin serum concentrations are not associated with underlying disease etiology. Box plot are displayed, where the bold line indicates the median per group, the box represents 50% of the values, and horizontal lines show minimum and maximum values of the calculated non-outlier values; asterisks and open circles indicate outlier values.

In rat models of polymicrobial sepsis induced by cecal ligation and puncture (CLP) as well as in a small pilot study with 16 critically ill surgical and medical patients, decreased circulating levels of ghrelin have been reported [18,21]. On the other hand, a later study proved significantly increased ghrelin concentrations in response to endotoxin administration in dogs [22]. To examine the significance of ghrelin in humans in a genuine intensive care environment we analyzed blood samples of critically ill patients at admission to a Medical ICU. As demonstrated in Figure 1a critical care patients had significantly higher serum ghrelin levels than healthy volunteers (median 9.6 pmol/L in controls vs. 18.4 pmol/L in patients, *P *<0.001). However, there was considerable overlap between controls and patients (Figure 1a). Ghrelin did not correlate with age or sex in either controls or patients (data not shown). The subgroup analysis of septic and non-septic patients showed no difference in ghrelin serum concentrations in both groups (Figure 1b), possibly indicating that critical illness by itself and not inflammation or endotoxemia is the primarily driving ghrelin elevation.

### Ghrelin serum concentrations in critically ill patients are not associated with underlying etiologies

We could previously demonstrate in patients with liver cirrhosis that ghrelin serum concentrations are not correlated with liver function, but are increased in advanced stages (for example, Child C cirrhosis) and in case of complications of chronic liver disease [23]. To test the impact of the underlying etiology of critical illness we performed extensive subgroup analysis. Therefore, non-sepsis patients were divided into liver cirrhosis and others (mostly cardiovascular disorders) and sepsis patients into pulmonary and abdominal site of infection (Figure 1c). We especially focused on the cohort of patients with abdominal sepsis to account for a suggested link to ghrelin levels, as it has been recently reported that ghrelin administration ameliorates sepsis-induced derangements of gut barrier function in animal models [13]. However, although we could demonstrate a trend to higher ghrelin serum concentrations in patients with liver cirrhosis, our findings did not reach statistical significance for the different etiological subgroups of *pulmonary sepsis*, *abdominal sepsis*, *liver cirrhosis *and *non sepsis *patients as displayed in Figure 1c.

### Ghrelin serum concentrations are not correlated with pre-existing diabetes mellitus or body mass index

**Figure 2:**
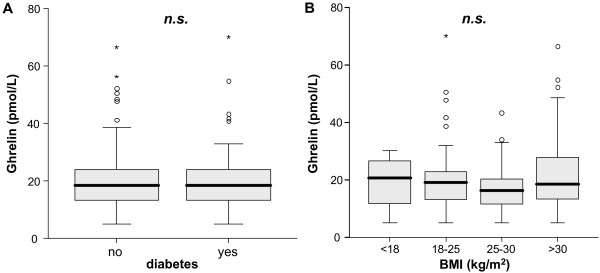
**Association of serum ghrelin with diabetes mellitus and body mass index in critically ill patients**. **(A) **Serum ghrelin levels do not differ between patients with (n = 56) or without (n = 114) pre-existing diabetes mellitus on admittance to the ICU. **(B) **In subgroup analysis of patients with BMI <18 (n = 5), BMI 18 to 25 (n = 63), BMI 25 to 30 (n = 43) and BMI >30 kg/m^2 ^(n = 40) no differences in serum ghrelin concentrations can be demonstrated. Box plot are displayed, where the bold line indicates the median per group, the box represents 50% of the values, and horizontal lines show minimum and maximum values of the calculated non-outlier values; asterisks and open circles indicate outlier values.

Stimulation of appetite and regulation of energy homeostasis have been identified as major functions of ghrelin. By these means ghrelin directly contributes to obesity [24]. It is therefore important to exclude that endogenous ghrelin serum levels found in ICU patients upon admission solely reflect their nutritional status. We therefore performed subgroup analyses to evaluate the effect of pre-existing diabetes mellitus and body mass index (BMI) on serum ghrelin levels, by comparing diabetic with non-diabetic as well as patients with BMI <18, BMI 18 to 25, BMI 25 to 30 and BMI >30 kg/m². No significant correlation between pre-existing diabetes mellitus or short weight, normal weight, overweight and obesity could be demonstrated (Figure 2a, b and data not shown).

### Ghrelin serum concentrations are not correlated to markers of inflammation or hepatic function in critically ill patients

In contrast to our findings (Figure 1), decreased levels of ghrelin in some rodent models of polymicrobial sepsis have been demonstrated [21,25]. Additionally, treatment with ghrelin in these sepsis models reduced serum concentrations of proinflammatory cytokines like TNF-α and IL-6 [12]. In our study we could not reproduce this possible link of inflammatory markers to ghrelin in the large cohort of critically ill patients. Indeed, TNF-α, IL-6, white cell blood count, C-reactive protein or procalcitonin did not correlate with serum ghrelin concentrations neither in all ICU-patients, nor in the subgroups of sepsis and non-sepsis patients in the clinical setting at admission to the Medical ICU (data not shown). With respect to organ function, we could not demonstrate a significant correlation between ghrelin and hepatic function as displayed by concentrations of serum protein, albumin, prothrombin time, antithrombin III and pseudocholinesterase activity (data not shown). Of note, ghrelin did not correlate with growth hormone or insulin-like growth factor-1 (IGF-1) serum levels either (data not shown).

### Ghrelin correlates inversely with renal function and markers of glucose metabolism in non-sepsis patients

**Figure 3:**
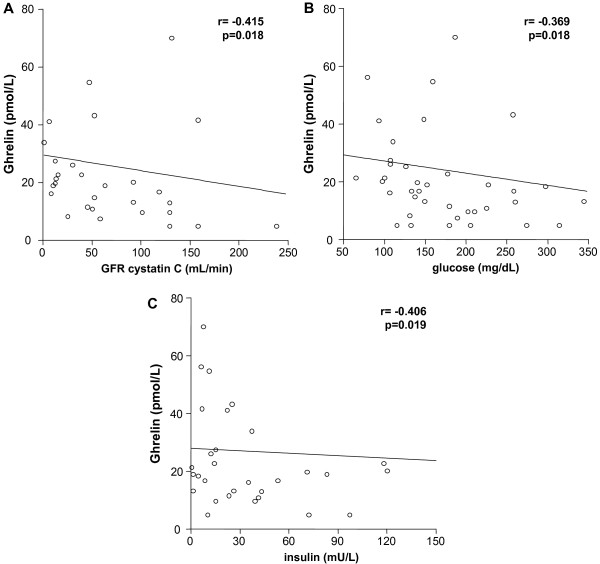
**Impact of organ dysfunction and markers of metabolism on serum ghrelin in non-sepsis patients**. **(A) **Serum ghrelin concentrations are elevated in non-sepsis ICU patients with renal failure, as demonstrated by a correlation with serum creatinine and an inverse correlation with the glomerular filtration rate (GFR, calculated using serum cystatin C measurements). **(B, C) **Markers of carbohydrate metabolism, such as glucose and insulin, are inversely correlated with serum ghrelin concentrations in non-sepsis ICU patients. Spearman rank correlation test, correlation coefficient *r *and *P*-values are given.

In total cohort of critically ill patients and the subgroup of septic patients no significant correlation between ghrelin serum concentrations and renal function (for example, glomerular filtration rate, cystatin C, creatinine) could be detected (data not shown). Surprisingly, ghrelin was inversely associated with renal function in the subgroup of non-sepsis patients as evidenced by significant correlations with the glomerular filtration rate of cystatin C (r = -0.415, *P *= 0.018; Figure 3a), indicating that reduced renal clearance might contribute to increased serum ghrelin in these patients. This suggests different regulatory mechanisms of ghrelin in critical illness either due to septic or non-septic etiologies. This hypothesis is further supported by our finding of a close inverse correlation of ghrelin with serum glucose (r = -0.369, *P *= 0.018) and insulin (r = -0.406, *P *= 0.019) as important markers of carbohydrate metabolism in non-sepsis patients at admission to the ICU (Figure 3b, c), but not in sepsis patients.

### High ghrelin levels indicate a favourable prognosis in sepsis patients

**Figure 4:**
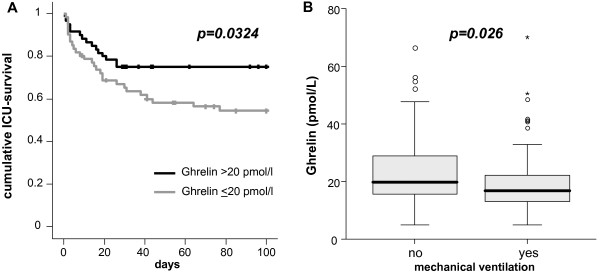
**Prognostic relevance of serum ghrelin in critically ill patients**. **(A) **Kaplan-Meier survival curves of ICU patients with sepsis (n = 122) are displayed, showing that sepsis patients with high ghrelin levels (>20 pmol/L, black) have a decreased short-term mortality at the ICU as compared to patients with low ghrelin (≤20 pmol/L, grey). *P*-value from Cox regression analysis is given. **(B) **For the total cohort of all critically ill patients (sepsis and non-sepsis), ghrelin serum concentrations at admission to the ICU were significantly higher if no mechanical ventilation was required (*P *= 0.026). Box plot are displayed, where the bold line indicates the median per group, the box represents 50% of the values, and horizontal lines show minimum and maximum values of the calculated non-outlier values; asterixes and open circles indicate outlier values.

To assess the impact of ghrelin on ICU- and overall-survival during a nearly three-year follow-up period among all critically ill patients and the subgroups of sepsis and non-sepsis patients we performed Cox regression analyses and used Kaplan-Meier curves. For the total cohort of all critical care patients, we could not demonstrate an association between survival and ghrelin serum levels using uni- and multivariate Cox regression analysis (data not shown). Likewise, ghrelin serum concentrations did not correlate with survival in non-sepsis patients (data not shown). Remarkably, high ghrelin serum concentrations upon admission to the Medical ICU were a predictor for a favourable prognosis concerning ICU-mortality in sepsis patients (*P *= 0.0324). Using a cut-off value for serum ghrelin of 20 pmol/L, Kaplan-Meier curves displayed significantly improved survival on the ICU for sepsis patients with high ghrelin (log rank 4.58). In line, surviving sepsis patients had significantly higher ghrelin serum concentrations (median 19.1 pmol/L) than non-survivors (median 16.3 pmol/L, *P *= 0.016, U-Test; Figure 4a).

### Ghrelin is significantly associated with the necessity of ventilation in critically ill patients

A protective effect of ghrelin on severe sepsis induced acute lung injury (ALI), mediated by inhibition of NF-κB-pathway in the lungs, has been demonstrated in animal models [14]. In order to possibly translate this protective effect of ghrelin on pulmonary function into critically ill patients in a medical ICU, ghrelin was correlated to the necessity of ventilation as an indirect marker of pulmonary function. Ghrelin serum concentrations at admission to ICU were significantly higher if no mechanical ventilation was required in the total cohort of all critically ill patients (*P *= 0.026) and in sepsis patients as well (*P *= 0.022; Figure 4b).

## Discussion

Contradictory findings on the responses of ghrelin to endotoxin in animal models have been reported, and data are not fully consistent [21,22]. Current data either demonstrated increased or decreased ghrelin concentrations after administration of endotoxin. In a rat model of cecal ligation and puncture (CLP) induced sepsis, significantly decreased ghrelin serum concentrations at early (5 h after CLP) and late (20 h after CLP) stages of sepsis were reported. In contrast, elevated ghrelin serum concentrations and a strong association of ghrelin to markers of inflammation and hepatic and renal function were observed in dogs after endotoxinaemia [22]. As a conclusion of this study, elevated ghrelin levels have been considered as an “adaptive protective response to endotoxin”. These findings are supported by a previous study in rats where serum ghrelin levels were found to be significantly increased upon endotoxin shock [26]. Furthermore, this study demonstrated a therapeutic effect of ghrelin infusion by the means of a significantly decreased mortality rate and ameliorated hypotension due to septic shock.

Little is known about the function of ghrelin in sepsis in humans. A study on healthy volunteers identified ghrelin as one of the first hormones increasing in the physiological response to endotoxinaemia [17]. There are no data on the profiles of circulating ghrelin levels in critically ill patients treated at a medical ICU. In a small study of 25 surgical patients with postoperative intraabdominal sepsis elevated ghrelin levels have been reported [27]. In contrast, a study of 16 surgical and non-surgical ICU patients showed significantly reduced serum ghrelin levels [18]. Before promoting ghrelin as a new therapeutic target in intensive care medicine it is (in our opinion) essential to elucidate the regulation of ghrelin in human critical illness, sepsis and septic shock from a clinical point of view. In the present study we can demonstrate for the first time in a large, well characterized cohort of patients from a medical ICU that ghrelin levels are significantly elevated in all critically ill patients as compared to healthy controls, albeit with a considerable overlap between both groups (Figure 1a). Ghrelin serum concentrations did not differ between sepsis and non-sepsis patients, which might indicate that high serum ghrelin levels rather reflect the impact of critical disease than being directly influenced by inflammatory cytokines in sepsis or septic shock (Figure 1b). According to this we could not demonstrate any correlation of ghrelin with *classical *markers of inflammation as white blood cell count, C-reactive protein, procalcitonin, TNF-α or IL-6.

Ghrelin stimulates physiologically growth hormone (GH) secretion independent of hypothalamic GH-releasing hormone and causes weight gain and obesity by increasing food intake and diminishing lipid utilisation in non-critically ill individuals [28,29]. Before a meal ghrelin serum levels rise and show an abrupt decline at the beginning of food intake with trough levels within one hour after eating [30]. Ghrelin serum levels are decreased in obese patients and elevated in patients with anorexia nervosa [31,32]. In our study population of critically ill patients we could not establish a significant correlation between ghrelin and the body mass index as a parameter reflecting the nutritional status upon admission to ICU. This observation strongly indicates that ghrelin regulation is not primarily driven by the current nutritional status, but by mechanisms related to the stress of critical disease.

In fact, the mechanisms of ghrelin release are not satisfyingly understood at present. The most important factor is food intake, but possibly blood glucose and insulin may participate in regulation [24]. However, we found a close inverse correlation of ghrelin with serum glucose and insulin only in the subgroup of non-sepsis patients (Figure 3b, c), but not in sepsis patients. It is therefore very likely that additional, so far not apparent factors in the complex and multifactorial metabolic disturbance in critically ill patients impact serum ghrelin. Similarly, the growth hormone and IGF-1 axis, both targets of physiological ghrelin effects, have been reported to be heavily deranged in ICU patients [33]. However, direct therapeutic intervention by administration of growth hormone in critically ill patients resulted in increased mortality [34]. This underlines that the changes of metabolism in critical illness and sepsis are complex, multifactorial, and future studies are warranted to unravel these interactions.

Furthermore, we could identify high ghrelin levels as a prognostic marker for survival at the ICU in sepsis patients (Figure 4). Assuming that high ghrelin levels have protective effects in sepsis, as demonstrated by ghrelin administration in several animal studies [11,13-15], our findings support the concept to view ghrelin upregulation as beneficial in severe sepsis and septic shock in humans. Several mechanisms may concertedly mediate the benefical effect of circulating ghrelin. Specifically, intravenous Ghrelin administration in healthy humans or animal studies has been found to reduce peripheral vascular resistance and increase cardiac output without a significant change in heart rate, resulting in improved tissue perfusion [11,35]. Ghrelin also exerted protective effects in an experimental model of acute, endotoxin-induced kidney failure [15]. Furthermore, ghrelin mediated protective effects on pulmonary function though inhibition of NF-κB in an animal model of acute lung injury [14]. Regarding the necessity of mechanical ventilation as a surrogate parameter of pulmonary function in patients, we could demonstrate significantly higher ghrelin serum concentrations in spontaneous breathing critically ill patients as compared with mechanically ventilated patients (Figure 4b). That might advert to pulmonary protective effects of high ghrelin serum concentrations in critically ill patients, keeping in mind that ghrelin regulation is most likely multifactorial and not fully understood.

## Conclusions

We could demonstrate, for the first time, high ghrelin levels in critically ill patients as compared to healthy controls, independent of the presence of sepsis or inflammatory markers. Moreover, high ghrelin levels were a positive predictor of ICU-survival in sepsis patients, matching previous results from animal models. Nevertheless, the regulation of cytokines, adipokines and hormones with metabolic functions in critical illness is complex, and both, future experimental and clinical studies are needed to identify and evaluate ghrelin as a potential new therapeutic agent in critical care medicine.

## Key messages

• Recent animal studies identified ghrelin, a stomach-derived ligand for the growth hormone receptor, as a mediator of various beneficial effects in sepsis.

• Ghrelin serum concentrations are significantly elevated in critically ill patients at admission to the ICU, but do not differ between sepsis and non-sepsis patients.

• High ghrelin levels indicate a favourable prognosis in sepsis patients.

• Low ghrelin is associated with the necessity of ventilation as a parameter of adverse pulmonary function in ICU patients, and serum ghrelin correlates with renal function in non-sepsis patients.

• Our data support the further investigation of ghrelin as a prognostic tool in ICU patients and its potential therapeutic application in sepsis.

## Abbreviations

ALI: acute lung injury; APACHE II: acute physiology and chronic health evaluation; BMI: body mass index; CLP: cecal ligation and puncture; CRP: C-reactive protein; ELISA: enzyme-linked immunosorbent assay; GFR: glomerular filtration rate; GH: growth hormone; GHS-R1a: growth hormone secretagogue receptor 1a; ICU: intensive care unit; IL-6: interleukin 6; IL-10: interleukin 10; IGF-1: insulin-like growth factor-1; p: p-value; PCHE, pseudocholinesterase; r: correlation coefficient; SAPS: simplified acute physiology score; SIRS: systemic inflammatory response syndrome; TNF-α: tumor necrosis factor α.

## Competing interests

The authors declare that they have no competing interests.

## Authors' contributions

AK, FT and CT designed the study, analyzed data and wrote the manuscript. ES, AH and SV collected data and assisted in patient recruitment.
